# Horner's Syndrome Following Dural Puncture Epidural Analgesia for Labor

**DOI:** 10.7759/cureus.48666

**Published:** 2023-11-11

**Authors:** Sadafsadat Mirkarimi, Shima Zargar, Sanaz Beig Zali, Benjamin Fuller

**Affiliations:** 1 Anesthesiology, University of Minnesota, Minneapolis, USA; 2 Neurology, University of Texas Health San Antonio, San Antonio, USA; 3 Anesthesiology, Tabriz University of Medical Sciences, Tabriz, IRN

**Keywords:** self limited, lumbar dural puncture, labor pain, labour epidural analgesia, horner’s syndrome

## Abstract

Horner's syndrome has been identified as an adverse outcome associated with the administration of epidural analgesia during labor. This syndrome is attributed to the upward spread of the local anesthetic, which may extend toward the superior cervical sympathetic chain. This process could disrupt the sympathetic pathways supplying the facial and ocular areas. We describe a case of a 26-year-old primigravid female with transient isolated Horner's syndrome following dural puncture epidural analgesia during labor.

## Introduction

Horner's syndrome (HS) is a result of the interruption of sympathetic innervation to ocular structures and is characterized by ipsilateral miosis, facial anhidrosis, enophthalmos, conjunctival injection, and facial flushing [1,2]. HS has been reported in connection with various regional anesthetic techniques, such as those involving the brachial plexus, cervical plexus, paravertebral, and epidural blockade [2,3]. This syndrome is associated with the blockade of oculosympathetic fibers, which occurs at the point where preganglionic neurons exit the spinal cord and then ascend through the sympathetic chain to reach the superior cervical ganglion [2].

Reports have indicated a heightened incidence of HS within the obstetric population, particularly among individuals undergoing epidural anesthesia for cesarean sections [2,4-6]. Therefore, we report a case with transient isolated HS to raise awareness about this condition as a rare but self-limited complication of labor epidural anesthesia. We also outline the precautions that must be considered during evaluation and management.

## Case presentation

An otherwise healthy 26-year-old primigravid female (104 kg, 180 cm, BMI 32.1) was admitted at 39 weeks of gestation in active labor and requested epidural analgesia for labor pain relief. Dural puncture epidural (DPE) analgesia was performed with the first attempt at the L3-L4 interspace with the patient in the sitting position using a loss of resistance to saline technique. A 17-gauge Tuohy needle was used to find the epidural space, and a Pencan® 25-gauge spinal needle (B. Braun, Melsungen, Germany) was used for dural puncture with a spontaneous return of cerebrospinal fluid (CSF). The epidural space was reached at a depth of 5.5 cm, and a 19-gauge epidural catheter was threaded easily with the needle pointing toward the cephalad and fixed at a depth of 10.5 cm in the skin. As per our institutional protocol, a test dose of 1.5% lidocaine with 1:200,000 epinephrine was administered after a negative aspiration test; 8 mL of 0.125% bupivacaine was administered manually, and a programmed intermittent epidural bolus (PIEB) of 0.1% ropivacaine with fentanyl (2 μg/mL) was started at the rate of 8 mL/h with patient-controlled epidural analgesia (PCEA) of 6 ml with a lockout period of 10 minutes for breakthrough pain. Ten minutes after initiating the epidural anesthesia, the patient reported satisfactory pain relief. A bilateral T7 sensory block level was noted on the exam, but the patient subjectively complained of mild heaviness in her right eyelid. The PIEB/PCEA was temporarily stopped. The patient’s vital signs remained unremarkable, continuous intrapartum fetal monitoring showed normal fetal heart rate (FHR) variability, and no other neurological findings were detected. Therefore, the epidural analgesia was resumed at the same rate, and subjective eye heaviness resolved within 15 minutes. After six hours, the patient reported decreased pain relief, with a sensory level at T10 bilaterally, and requested supplemental analgesia. Following a negative aspiration, a 10 mL dose of 0.125% bupivacaine was administered while the patient was in a sitting position, providing satisfactory labor analgesia within 20 minutes. However, the patient exhibited right-sided partial ptosis, miosis, facial flushing, and ipsilateral conjunctival hyperemia without a motor blockade.

The sensory block was at the T7 dermatome bilaterally. The patient denied difficulty breathing or weakness in the upper extremities. No other neurological findings were detected. Vital signs remained stable throughout the entire time. Fetal monitoring showed normal FHR variability. As evident in our case, the patient's symptoms were most prominent after receiving the epidural top-up injection. To address this, the case was managed by implementing a strategic approach that involved reducing the epidural PIEB rate, offering reassurance to the parturient, and refraining from administering any additional boluses either manually or PCEA until the time of delivery. The patient had a complete resolution of her symptoms within 20 minutes after delivery and 75 minutes after the last bolus administration. The rest of her hospital course and six-month follow-up remained asymptomatic.

## Discussion

Literature has reported HS as a benign, rare, and underreported complication of labor epidural analgesia [2]. It seems more common in the obstetric population than in the general population, possibly due to anatomic and physiologic changes in pregnancy. Rabinovich conducted a comprehensive study involving 4598 parturients who underwent lumbar epidural analgesia, revealing an incidence rate of 0.13% for the occurrence of HS [6]. However, this number was found to significantly increase to 4% when utilizing an epidural top-up for cesarean section [7].

A retrospective review of 78 case reports in 2018 revealed that most patients presented with unilateral miosis and ptosis, developing within one hour of a local anesthetic bolus and resolved within a median of two hours [2]. However, cases with bilateral symptoms accompanying trigeminal and hypoglossal nerve palsy, maternal hypotension, and fetal bradycardia have also been reported [8]. High cephalad spread of local anesthetics has been found to be responsible for transient HS after epidural analgesia, indicating a high-level block interrupting the oculosympathetic pathway [2,9]. 

Historically, a correlation has been observed between higher levels of epidural block with high BMI, increased distance between the skin and epidural space (4-8 cm), the type and dosage of local anesthetics administered, and the lateral decubitus positioning [6]. Biousse suggested that local anesthetics’ distribution within the epidural space might be affected by gravity and positioning. This could result in HS appearing more prominently on the dependent side when a patient is in a lateral decubitus position during the epidural placement or when an anesthetic solution is administered [4]. 

Rabinovich et al. reported no association between BMI, position during epidural injection, catheter length, and epidural solution type and concentration as risk factors for HS. They observed that in three of the four patients whose procedures were performed in the decubitus position, HS was noted on the contralateral side. However, they confirmed the epidural top-up technique's influence on HS incidence [6]. 

Chandrasekhar and colleagues reported a case of unilateral HS during lumbar epidural analgesia for labor, in which a 0.04% bupivacaine concentration was used, suggesting that even a diluted local anesthetic may lead to the development of HS [10]. The condition is generally transient, self-limited, has a favorable prognosis, and does not warrant further extensive investigation; however, clinical monitoring of cardiorespiratory and neurological symptoms, as well as cranial nerve involvement, is essential for high sympathetic blocks that have the potential to cause cardiovascular compromise [2]. It must be noted that maternal hypotension can also cause transient fetal bradycardia [6].

Two explanations have been proposed for the etiology of the high cephalad spread of local anesthetics leading to HS during labor: anatomic variations and subdural blockade [11]. Patients have wide variations in epidural anatomy. Anatomical variations that facilitate the ascension of local anesthetic are the presence of fibrous septae in the epidural space, spinal deformities, post-surgical adhesions, and repeated epidural punctures [2,11]. In pregnant individuals, several anatomical and physiological changes have been believed to favor the cephalic spread of the epidural solution. These include increased intra-abdominal pressure due to gravid uterus, uterine contractions, the engorgement of the epidural venous plexus, which reduces the epidural space volume, and elevated progesterone levels that enhance the sensitivity of neuronal membranes to local anesthetics and facilitates the disruption of sympathetic fibers while potentially sparing sensory and motor fibers [2,9]. Inadvertent subdural injection of a low-dose local anesthetic has been suggested as another possible etiology for HS development. Therefore, it should be assessed via epidurography in select patients [4,6]. 

Misplacement of the catheter within the subdural space, which communicates with the intracranial space, can lead to the spreading of local anesthetics to intracranial structures, consequently causing life-threatening complications such as cardiorespiratory and neurological compromise [11,12]. Mehta and Salmon, through radiographic imaging, observed that subdural cannulation happens in 7% of all planned epidural catheter insertions [13]. Turbelin et al. suggested that misplacement of the catheter within the subdural space caused a recurrence of HS three times in the same parturient during labor [12].

Bernards' study has illuminated that local anesthetics tend to follow the pressure gradient, simplifying their transit from the epidural space into the subarachnoid space through the puncture hole [14]. The flow between the epidural and dural spaces is influenced by the size of the needle. Using a larger spinal needle, as suggested in Cappiello's study, would facilitate a more significant transfer of epidurally administered analgesic doses to the subarachnoid space [15].

In our case, we utilized a combination of DPE with PIEB technology to enhance the bolus pressure, which subsequently increased the transfer rate of epidurally administered medication to the subarachnoid space. Our hypothesis was that this might have elevated the likelihood of causing HS. To the best of our knowledge, there is currently no reported case of HS resulting from the use of the DPE technique in epidural procedures. Further investigations are only warranted if there are additional or persistent neurological deficits or complicating factors. Complete resolution of neurological symptoms should be confirmed post delivery. Persistent HS or atypical features, such as neck pain, warrant additional diagnostic workup [2].

## Conclusions

HS associated with epidural analgesia in labor is rare, primarily transient, and has a favorable prognosis. However, it is prudent to consider the exclusion of other potentially life-threatening complications. Despite its generally benign prognosis, anesthesia providers should be aware that the cephalic spread of local anesthetic could serve as an indicator of a high sympathetic blockade. Furthermore, by raising awareness among anesthesiologists, this proactive approach can enhance management effectiveness and reduce the need for costly and unnecessary diagnostic investigations.
